# How to Vulcanize Rubber Plates between Metallic Surfaces

**Published:** 1895-10

**Authors:** A. N. Dick

**Affiliations:** Woodland, Cal.


ARTICLE VI.

### HOW TO VULCANIZE RUBBER PLATES BETWEEN METALLIC SURFACES.

BY DR. A. N. DICK, WOODLAND, CAL.

The process which I employ is the following: After the teeth have been articulated, and the model buried in the lower half of the flask, trim away all surplus wax from the palatal side of the teeth, leaving the model exposed.

Now take a sheet of good modeling compound, rolled to the thickness desired for the plate, dip it in hot water and adjust it to the model and teeth, using a spoon to pour on the hot water till it fits perfectly, so as to develop the ruge on the lingual surface. Then with a sharp instrument trim the edges and finish to the desired shape of plate. Moisten the surface and burnish on a piece of extra tough tin foil No. 4, first pressing it to position with a bunch of cotton on the corner of a soft napkin. Let the burnishing be done very carefully with a smooth round instrument.

I prepare my sheet of modeling compound by pressing it between two pieces of glass. In that way I secure any thickness that I desire.

In preparing the plaster for the mold, pour the required amount of water in the mixing cup and sprinkle the plaster on the water without stirring, till enough of the plaster has settled down in the water to give the desired consistency; by so doing, the air that is in the dry plaster will be floated out, so to speak, and not be carried in the mixture as it would be if the plaster was stirred from the beginning, thus securing a mold free from air bubbles. Then pour and let it set.

After opening the flask, pour a little hot water on the base-plate and lift it carefully from the underlying tin foil

If these details are strictly followed by a skillful hand, the result will be a beautiful lingual surface that will re quire only the felt and brush wheels to finish it. The file and sandpaper will be needed for the margins.

To secure a metallic surface for the palatal side of the plate is equally simple, and, I think, to the mind of the practical man, needs no explanation. However, I would suggest that the same care should be used in mixing the plaster for the model as for the mold, otherwise there would be no air holes in which the rubber would be forced through the tin foil, and thus make a rough surface. The foil for the palatal side should be No. 4, and the model should be wet when the foil is applied to it. The foil should be applied to the model with the thumb and fingers without burnishing, as the burnisher would injure the model.—*Items of Interest.*

